# The Validation of the Greulich and Pyle Atlas for Radiological Bone Age Assessments in a Pediatric Population from the Canary Islands

**DOI:** 10.3390/healthcare12181847

**Published:** 2024-09-14

**Authors:** Isidro Miguel Martín Pérez, Sebastián Eustaquio Martín Pérez, Jesús María Vega González, Ruth Molina Suárez, Alfonso Miguel García Hernández, Fidel Rodríguez Hernández, Mario Herrera Pérez

**Affiliations:** 1 Escuela de Doctorado y Estudios de Posgrado, Universidad de La Laguna, San Cristóbal de La Laguna, 38203 Santa Cruz de Tenerife, Spain; alu0100705157@ull.edu.es (S.E.M.P.); almigar@ull.es (A.M.G.H.); 2 Departamento de Farmacología y Medicina Física, Área de Radiología y Medicina Física, Sección de Enfermería y Fisioterapia, Facultad de Ciencias de la Salud, Universidad de La Laguna, 38200 Santa Cruz de Tenerife, Spain; frguezh@ull.edu.es; 3 Musculoskeletal Pain and Motor Control Research Group, Faculty of Health Sciences, Universidad Europea de Canarias, 38300 Santa Cruz de Tenerife, Spain; 4 Musculoskeletal Pain and Motor Control Research Group, Faculty of Sport Sciences, Universidad Europea de Madrid, 28670 Villaviciosa de Odón, Spain; 5 Institute of Legal Medicine and Forensic Sciences of Santa Cruz de Tenerife, 38230 San Cristóbal de La Laguna, Spain; jveggonf@justiciaencanarias.org; 6 Pediatric Endocrinology Unit, Pediatric Department, Hospital Universitario de Canarias, San Cristóbal de La Laguna, 38320 Santa Cruz de Tenerife, Spain; rmolsua@gobiernodecanarias.org; 7 School of Medicine (Health Sciences), Universidad de La Laguna, 38200 Santa Cruz de Tenerife, Spain; mherrera@ull.es; 8 Foot and Ankle Unit, Orthopedic Surgery and Traumatology Department, San Cristóbal de La Laguna, 38320 Santa Cruz de Tenerife, Spain

**Keywords:** diagnostic imaging, radiography, age determination by skeleton, children, Greulich and Pyle Atlas, Canary Islands

## Abstract

Bone age assessments measure the growth and development of children and adolescents by evaluating their skeletal maturity, which is influenced by various factors like heredity, ethnicity, culture, and nutrition. The clinical standards for this assessment should be up to date and appropriate for the specific population being studied. This study validates the GP-Canary Atlas for accurately predicting bone age by analyzing posteroanterior left hand and wrist radiographs of healthy children (80 females and 134 males) from the Canary Islands across various developmental stages and genders. We found strong intra-rater reliability among all three raters, with Raters 1 and 2 indicating very high consistency (intra-class coefficients = 0.990 to 0.996) and Rater 3 displaying slightly lower but still strong reliability (intra-class coefficients = 0.921 to 0.976). The inter-rater agreement was excellent between Raters 1 and 2 but significantly lower between Rater 3 and the other two raters, with intra-class coefficients of 0.408 and 0.463 for Rater 1 and 0.327 and 0.509 for Rater 2. The accuracy analysis revealed a substantial underestimation of bone age compared to chronological age for preschool- (mean difference = 17.036 months; *p* < 0.001) and school-age males (mean difference = 13.298 months; *p* < 0.001). However, this was not observed in females, where the mean difference was minimal (3.949 months; *p* < 0.239). In contrast, the Atlas showed greater accuracy for teenagers, showing only a slight overestimation (mean difference = 3.159 months; *p* = 0.823). In conclusion, the GP-Canary Atlas demonstrates overall precision but requires caution as it underestimates the BA in preschool children and overestimates it in school-age girls and adolescents.

## 1. Introduction

Maturation encompasses the physical and psychological development that occurs from childhood to adulthood [1,2,3]. Key indicators of biological maturation include sexual maturity [4,5,6], skeletal maturity [7,8,9], and morphological maturity [10]. Skeletal maturity is determined by a combination of genetic and environmental factors [11,12]. In optimal conditions, genetic factors account for approximately 80% to 90% of the maturation process; however, in less favorable environments, their influence can decrease to about 60% [13,14]. Various methods are available for assessing skeletal maturity [15,16,17,18], with radiographic analyses being among the most widely used [19]. One of the most common techniques for assessing skeletal maturity and predicting growth potential is the radiological evaluation of bone age (BA) using the Greulich and Pyle Atlas (GP Atlas) [11,20]. This method involves comparing the radiographic appearance of bones to standardized maturity levels for specific chronological age (CA) groups [21].

The BA is influenced by a range of biological and socio-cultural factors [22], including genetics, nutrition, socioeconomic status, and overall health. These factors can vary widely across different populations, leading to significant differences in bone maturation [23]. As a result, the normative data used in clinical practice must be current and specific to the population being assessed to ensure accurate evaluations [24]. The GP Atlas involves comparing children’s posteroanterior left hand and wrist radiographs (PA-HW) to reference plates created from a study of white upper-middle-class children conducted between 1932 and 1942 [19]. This atlas has been validated for use in various ethnic groups [25]. However, a recent systematic review indicated that, while the GP Atlas is generally considered reliable, its accuracy can vary and is not always consistent [26]. This inconsistency is particularly evident in the GP Atlas’s tendency to underestimate the BA in younger Caucasian and Hispanic populations, which may result in misinterpretations and potentially impact clinical decision making [27].

The Canary Islands’ (Spain) diverse genetic composition, influenced by indigenous Guanche ancestry, European settlers, and African and American admixture [28], combined with distinct environmental factors could lead to variations in bone development. Furthermore, the archipelago’s geographical isolation and specific socio-cultural practices might also contribute to differences in growth patterns and maturation rates compared to other populations. As a result, the GP Atlas may not provide a precise and accurate BA for this pediatric population, suggesting that its applicability may be limited [29]. Therefore, to mitigate the potential biases associated with using the original GP Atlas in the pediatric population, a region-specific adaptation, the Radiological Reference Atlas for Bone Age in the Canary Islands Population (GP-Canary Atlas) [2], was launched in 2009, which compiles data from 1978 [24] and has since become the standard reference for pediatricians in the region.

Although the GP-Canary Atlas was developed to enhance BA assessments for the pediatric population of the Canary Islands, it presents several methodological limitations. These include the use of a single set of radiographic images without gender differentiation, an inadequate selection of specific radiographs corresponding to different age stages, and the absence of formal validation procedures. These shortcomings raise concerns regarding its validity for BA determinations and underscore the need for a comprehensive evaluation to confirm the Atlas’s applicability in this population. Therefore, this study aims to assess the precision of the GP-Canary Atlas through intra-rater reliability and an inter-rater agreement analysis, as well as to determine its accuracy and explore potential differences in estimations based on developmental stages and gender.

## 2. Materials and Methods

### 2.1. Study Design

A cross-sectional study was undertaken between 1 September 2023, and 20 June 2024 within the Departments of Pediatrics and Orthopaedic and Trauma Surgery at Complejo Hospitalario Universitario de Canarias, a tertiary-level referral healthcare center in Tenerife, Spain. This study adhered to the STARD 2015 [30], which provides an updated checklist for reporting diagnostic accuracy studies, to ensure rigorous methodological and reporting standards. Ethical clearance was obtained from the Ethics Committee of Complejo Hospitalario Universitario de Canarias (reference number CHUC_2023_86, approved on 13 July 2023). The study protocol was strictly in compliance with the ethical principles outlined in the Declaration of Helsinki.

In order to provide detailed contextual data for the analysis, sociodemographic variables—including age and gender—as well as anthropometric measurements such as height, weight, and body mass index (BMI) were carefully extracted from the SAP Logon database (IBM^®^, Armonk, NY, USA) at the Complejo Hospitalario Universitario de Canarias (Tenerife, Canary Islands, Spain). In addition, standardized PA-HW radiographs, which were securely stored in the Centricity PACS system (GE HealthCare^®^, Chicago, IL, USA) at the same institution, were systematically analyzed for all participants. This was carried out according to a rigorously predefined protocol designed to ensure both consistency and precision in data collection and interpretation.

As a part of this verification process, each PA-HW radiograph was meticulously reviewed to confirm that the patient’s left hand was correctly positioned, with the fingers slightly spread and the wrist properly aligned with the forearm [31,32]. Moreover, the radiographs were carefully examined to ensure that all necessary anatomical landmarks, such as the phalanges, metacarpals, carpal bones, and distal radius and ulna, were clearly visible and appropriately captured. Additionally, the imaging settings—including exposure, focus, and contrast—were thoroughly checked to confirm strict adherence to the established protocol standards.

### 2.2. Participants

#### 2.2.1. Inclusion and Exclusion Criteria

The inclusion and exclusion criteria for this study were carefully defined to ensure a representative and homogeneous sample of healthy children and adolescents from the Canary Islands, enabling a precise assessment of BA using PA-HW radiographs. To be eligible for inclusion, (1) participants had to be healthy children aged 0 to 18 years who were long-term residents of the Canary Islands, defined as having resided there for a minimum of 5 years. Moreover, (2) at least one parent had to be of Canary Island origin, as verified through detailed medical and family history records, to ensure uniformity in the genetic background of the study population. Additionally, it was required that (3) subjects have medical records from 2016 onwards and that (4) their PA-HW radiographs adhered to predefined quality standards, including correct hand positioning, clear visibility of key anatomical landmarks, and compliance with standardized imaging protocols.

The exclusion criteria were designed to eliminate any confounding factors that could affect normal bone maturation or impede the accuracy of BA estimation. Participants were excluded if they had (1) medical conditions known to alter bone development, such as endocrine–metabolic disorders (e.g., growth hormone deficiency, hypothyroidism, or hyperthyroidism), neurological conditions (e.g., cerebral palsy or muscular dystrophy), or inherited disorders (e.g., Down syndrome, Turner syndrome, or Marfan syndrome). Furthermore, (2) children undergoing medical treatments that could influence skeletal growth (e.g., growth hormone therapy, corticosteroids, or chemotherapy) were excluded from the study. PA-HW radiographs were also excluded if they demonstrated (3) fractures, significant skeletal abnormalities, or (4) were of poor quality, characterized by an inadequate resolution, improper exposure, or obscured anatomical landmarks, which could interfere with the BA assessment procedure.

#### 2.2.2. Sample Size Calculation

The sample size for this study was calculated to ensure precise and accurate BA measurements using the GP-Canary Atlas, with a 95% confidence level and a 5% margin of error. Due to the lack of standard deviation data for BA in the Canary Islands’ population, estimates from similar studies in comparable populations were used to approximate expected variability [33,34,35]. Based on these estimates, a minimum of a total sample size of 100 subjects was determined to be sufficient for validating the GP Atlas in this pediatric population, ensuring reliable and generalizable findings.

### 2.3. Test Methods

During the evaluation process, three blinded raters independently assessed the PA-HW radiographs to ensure objective and unbiased BA determination. The raters included a radiology expert (Rater 1), a general practitioner (Rater 2) and a medical student (Rater 3), representing different levels of expertise and training in radiological interpretation. This diversity in raters was deliberately chosen to evaluate the impact of professional experience and training on the accuracy and consistency of BA assessments, thereby providing insights into the generalizability and robustness of the BA estimation method using the GP-Canary Atlas [2]. Each rater evaluated the radiographs by comparing the observed skeletal features with reference images from the GP-Canary Atlas. They used maturity indicators, such as ossification and bone fusion, to estimate the BA. If there was no exact match, the BA was estimated by averaging the ages of two consecutive radiographs from the Atlas [2].

To assess intra-rater precision, each rater determined BA at two distinct time points, T1 and T2, separated by less than one and a half months. The PA-HW radiographs were presented in a randomized and blinded sequence during both evaluations to minimize interpretation bias and prevent recall of previous assessments. This method allowed for a reliable examination of intra-rater reliability by comparing the BA measurements from T1 with those from T2 for each rater, thereby assessing the consistency of each evaluator’s assessments over time. With respect to inter-rater precision, the BA determinations were compared across the three raters to analyze the levels of agreement and reliability among different evaluators when interpreting the same set of PA-HW radiographs. This comparison was crucial to determine the reproducibility of the BA assessment method across raters with varying levels of expertise. Additionally, accuracy was determined by comparing the subjects’ CA, calculated from the difference between their birth date and the date of the radiological exam, with their estimated BA through the GP-Canary Atlas.

### 2.4. Analysis

Statistical analyses were conducted using IBM^®^ SPSS Statistics 29.0.1.0 software (Armonk, NY, USA). Descriptive statistics were first calculated for age (in mos.), weight (kg), height (m), and body mass index (BMI) (kg/m^2^). The data were stratified according to developmental stages as defined by Fraga and Fernández (2014) [36]—preschool children (1 to 5 years), school-age children (5 to 12 years), and teenagers (12 to 18 years)—and further segmented by gender to account for potential differences in bone maturation between males and females. These descriptive measures included calculations of central tendency (mean) and dispersion (standard deviation, minimum, and maximum) for both CA and BA as estimated by the study’s method. To confirm the suitability of the data for further statistical analyses, the Shapiro–Wilk test was applied to assess the normality of the data distribution, while Levene’s test was used to evaluate homoscedasticity.

For precision assessment, the intra-class correlation coefficient (ICC) was calculated to evaluate both intra-rater and inter-rater agreement. The ICC provided a robust quantitative measure of consistency within and between raters, indicating the degree of agreement when using the GP-Canary Atlas for BA estimation. Bland–Altman plots were also constructed to visually assess inter-rater reliability and detect any systematic bias or limits of agreement between the raters’ BA measurements. Moreover, the accuracy of the BA estimations was evaluated through a mean difference analysis, comparing the discrepancies between the estimated BA and the actual CA of the children.

## 3. Results

### 3.1. Characteristics of Sample

A total of 214 PA-HW radiographs from healthy children were finally included, consisting of 80 females and 134 males. In the preschool group, females had an average age of 39.33 mos. (SD = 15.18), an average weight of 14.52 kg (SD = 2.05), and an average height of 0.91 m (SD = 0.07), while males had an average age of 46.49 mos. (SD = 13.33), an average weight of 13.09 kg (SD = 2.17), and an average height of 0.94 m (SD = 0.05). In the school-age group, females averaged 92.00 mos. in age (SD = 26.08), 29.58 kg in weight (SD = 7.14), and 1.14 m in height (SD = 0.07), whereas males averaged 100.16 mos. in age (SD = 20.33), 23.67 kg in weight (SD = 4.85), and 1.16 m in height (SD = 0.05). In the teenager group, females and males both averaged 1.33 m in height, with females having an average age of 144.17 mos. (SD = 23.81) and average weight of 33.84 kg (SD = 4.62), while males had an average age of 151.53 mos. (SD = 20.17) and average weight of 34.21 kg (SD = 3.19). The Shapiro–Wilk test confirmed that all variables were normally distributed across these groups. More details are shown in Table 1.

### 3.2. Main Results

#### 3.2.1. Precision

- Intra-rater agreement

The ICC indicated strong precision and consistency in intra-rater reliability across all three raters when assessing the BA using the GP-Canary Atlas, with minor variations between genders. Specifically, Rater 1 showed high consistency, with an ICC of 0.995 (95% CI: 0.990–0.998) for females and 0.996 (95% CI: 0.992–0.998) for males. Similarly, Rater 2 demonstrated strong reliability, with an ICC of 0.990 (95% CI: 0.979–0.995) for females and 0.992 (95% CI: 0.982–0.996) for males. In contrast, Rater 3 reported slightly lower but still strong ICCs, with a value of 0.921 (95% CI: 0.832–0.964) for females and 0.976 (95% CI: 0.947–0.989) for males. More details are shown in Table 2.

- Inter-rater agreement

The inter-rater agreement in determining the BA using the GP-Canary Atlas showed notable differences between the female and male participants. For females, there was excellent agreement between Rater 1 and Rater 2, with an ICC of 0.982 (95% CI: 0.968, 0.990). However, the agreement was significantly lower between Rater 1 and Rater 3 and between Rater 2 and Rater 3, with ICCs of 0.463 (95% CI: 0.216, 0.654) and 0.509 (95% CI: 0.273, 0.688), respectively. For males, Rater 1 and Rater 2 demonstrated strong consistency with an ICC of 0.944 (95% CI: 0.902, 0.968). In contrast, the agreements between Rater 1 and Rater 3 and between Rater 2 and Rater 3 were lower, with ICCs of 0.408 (95% CI: 0.145, 0.618) and 0.327 (95% CI: 0.052, 0.557), respectively. These findings suggest that while there is high agreement between trained and general practitioner radiologists, the lower agreement with the student emphasizes the need for standardized training for evaluators using the GP-Canary Atlas. Further details can be found in Table 3.

**Figure 1:**
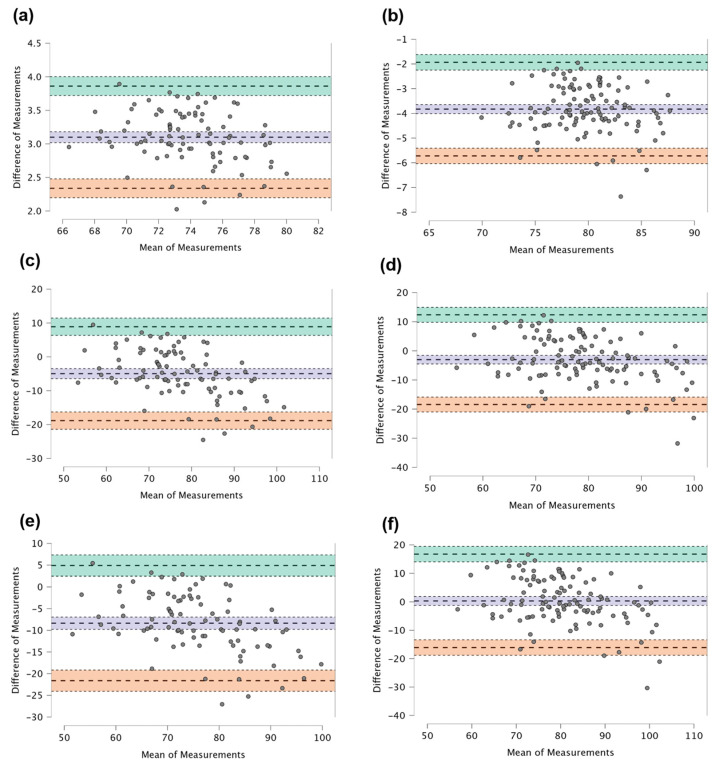
Bland–Altman plots illustrating BA assessments using the GP-Canary Atlas. The plots compare the assessments of Rater 1 with Rater 2 for both females (**a**) and males (**b**), Rater 1 with Rater 3 for females (**c**) and males (**d**), and Rater 2 with Rater 3 for females (**e**) and males (**f**). The dashed lines represent the mean differences, while the shaded areas in orange and green show the limits of agreement (±1.96 standard deviations). The purple lines represent the confidence intervals for the limits of agreement.

The Bland–Altman plots in Figure 1 illustrate the agreement among the three raters (Rater 1, Rater 2, and Rater 3) for the BA assessment using the GP-Canary Atlas. For female participants, Rater 1 and Rater 2 showed high agreement with a narrow range of differences, indicating strong consistency. In contrast, the agreements between Rater 1 and Rater 3 and between Rater 2 and Rater 3 were moderate with wider limits of agreement, suggesting more variability due to differences in training. A similar pattern was observed for male participants: Rater 1 and Rater 2 demonstrated strong agreement, while Rater 1 and Rater 3, and especially Rater 2 and Rater 3, exhibited lower agreement with broader ranges in differences, highlighting the challenges of achieving consistent assessments among less experienced raters.

#### 3.2.2. Accuracy

**Figure 2:**
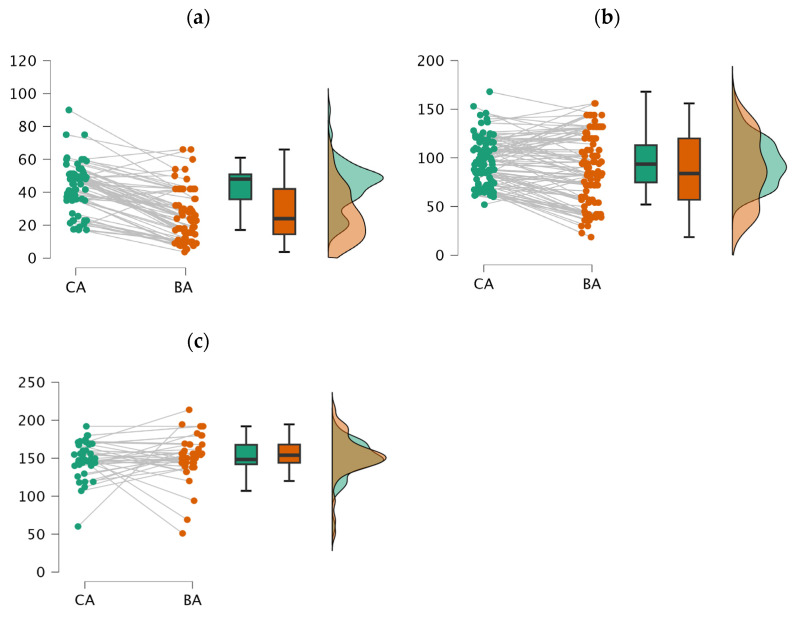
Accuracy of BA determination using GP-Canary Atlas across different developmental stages. Raincloud plots display BA accuracy in (**a**) preschool (1 to 5 years), (**b**) school-age (5 to 12 years), and (**c**) teenager (12 to 18 years) groups. Method shows significant BA underestimation and variability in preschool and school-age groups, while accuracy improves in teenager group with no significant differences between BA and CA.

The GP-Canary Atlas assessment method demonstrated a lack of accuracy in estimating the BA compared to the CA in both the preschool and school-age groups. Specifically, in the preschool group (ages > 1 to 5 years), the method significantly underestimated the BA with a mean difference (MD) of 17.036 mos. (*p* < 0.001). This underestimation was even more pronounced in females (MD = 15.081 mos., *p* < 0.001) than in males (MD = 14.898 mos., *p* < 0.001). Similarly, in the school-age group (ages > 5 to 12 years), the Atlas continued to underestimate the BA, although to a lesser extent, with an MD of 8.165 mos. (*p* < 0.001). Notably, the underestimation was more significant in males (MD = 13.298 mos., *p* < 0.001) compared to females (MD = 3.949 mos., *p* = 0.239). In contrast, the GP-Canary Atlas showed the highest accuracy in the teenage group (ages > 12 to 18 years), with only a slight overestimation of the BA (MD = 3.159 mos., *p* = 0.823). Interestingly, this overestimation was more pronounced in females (MD = 4.497 mos., *p* = 0.980) than in males (MD = 4.85 mos., *p* = 0.094). More details are provided in Table 4 and visually summarized in Figure 2.

## 4. Discussion

### 4.1. Precision of GP-Canary Atlas

#### 4.1.1. Intra-Rater Agreement

Our results show that the GP-Canary Atlas exhibits high intra-rater precision in BA assessments. The ICCs for evaluations by the radiology specialist (Rater 1) was nearly perfect, with an ICC of 0.995 for females and 0.996 for males. The general practitioner (Rater 2) also demonstrated high precision, with an ICC of 0.990 for females and 0.992 for males. However, the medical student (Rater 3) showed slightly lower precision, with an ICC of 0.921 for females and 0.976 for males.

These findings align with previous research in pediatric populations from Anglo-Saxon countries. Hackman and Black (2012) [37] reported an ICC of 0.969 for Scottish children, and Maggio et al. (2016) [38] found an ICC of 0.970 for males and 0.972 for females in Australia. Similarly, high correlations were reported in Germany and the Netherlands, with Schmidt et al. (2007) [39] finding an ICC of 0.96 for both genders and Van Rijn et al. (2001) [40] reporting an ICC of 0.979 for males and 0.974 for females. Our results also slightly exceed those reported in Southern European countries. Santos et al. (2011) [41] observed excellent intra-rater agreement in Portugal, with an ICC of 0.99 for both boys and girls, while Pinchi et al. (2014) [42] reported an ICC of 0.907 for males and 0.928 for females in Italy. However, Santoro et al. (2012) [43] found moderate intra-rater concordance in Southern Italy, with an ICC of 0.88 for males and 0.81 for females. Studies in the United States and Sweden have shown lower reliability, with Calfee et al. (2010) [44] reporting an ICC of 0.890 in a Latin American sample and Kullman (1995) [45] finding ICCs ranging from 0.64 to 0.74 in Swedish teenagers.

Similar high intra-rater agreements have been observed in African studies. Govender and Goodier (2018) [33] in South Africa reported an ICC of 0.99, and Olaotse et al. (2023) [46] in Botswana found an ICC of 0.97 for males and 0.98 for females. Dembetembe et al. (2012) [47] observed moderate precision (r = 0.76) using the GP Atlas in Cape Town. Comparable agreements have been reported elsewhere, such as in Saudi Arabia, with Albaker et al. (2021) [48] finding an ICC of 0.995 for males and 0.996 for females, and in Malaysia, with Nang et al. (2023) [49] reporting an ICC of 0.947 for males and 0.933 for females.

The excellent intra-rater agreement observed with both the GP-Canary Atlas and the GP Atlas can be attributed to the quick and direct visual comparisons they allow, facilitating efficient BA assessments across various pediatric populations. However, the slight variability observed when applying the GP-Canary Atlas may be due to individual cognitive biases and potential misinterpretations of the Atlas [50,51]. Biases such as anchoring, confirmation bias, experience-based bias, overconfidence, the availability heuristic, and the observer expectancy effect can impact the rater’s judgment, resulting in inconsistencies and longer times for BA assessments [52,53]. Additionally, errors may occur due to the limited number of maturity indicators available for evaluation, especially when assessing young children. As a child grows, the number of ossification points increases, but when fewer points are present, the potential for assessment errors also becomes higher.

#### 4.1.2. Inter-Rater Agreement

Our findings demonstrate a high level of agreement among different raters when using the GP-Canary Atlas for BA determinations. The concordance between the radiology specialist (Rater 1) and the general practitioner (Rater 2) was remarkably high for both women (ICC = 0.982) and men (ICC = 0.944), indicating that both raters consistently produced similar BA assessments.

On the one hand, these results align with those of previous studies on the degree of agreement between two expert evaluators when determining the BA of children from Anglo-Saxon countries. For instance, Alshamrani et al. (2019) [54] observed high agreement between two raters in a sample of British children aged 8.80 to 9.59 years. In Northern Europe, Zabet et al. (2015) [55] identified an excellent level of inter-rater concordance among assessors in France (ICC = 0.94; 95% CI: 0.91–0.96; *p* < 0.05). Similarly, Calfee et al. (2010) [44] found very high inter-rater reliability (ICC = 0.982) in a study involving children from Washington, United States. Additionally, significant agreement among examiners was reported in Oceania, with a Cohen’s kappa of 0.887 (*p* < 0.001) when the GP Atlas was used to assess BA in Western Australian children [38]. On the other hand, in Africa, there was a remarkable similarity between the GP-Canary Atlas and GP Atlas inter-rater agreement. Olaotse et al. (2023) [46] reported that the degree of agreement between two expert raters in assessing the BA in the Palapye region of Botswana reached an ICC of 0.94 for girls and 0.93 for boys.

However, the inter-rater reliability significantly declines when comparing the scores assigned by Rater 1 and Rater 3, as well as Rater 2 and Rater 3. This results in a noticeable reduction in agreement for both girls (ICC = 0.463 and 0.509, respectively) and boys (ICC = 0.408 and 0.327, respectively). The significant decrease in concordance among evaluators with varying levels of experience suggests that less experienced raters might interpret the characteristics of the images differently or make errors when applying the scoring criteria of the GP-Canary Atlas. As reported in other radiological diagnostic tests, this lack of precision may be due to limited familiarity with the specific methodology [56,57,58,59]. This highlights the need for more comprehensive training and rigorous standardization in evaluation procedures to ensure that all raters, regardless of experience, apply the criteria consistently and accurately. Such measures are crucial for preventing inconsistent diagnostic decisions in clinical practice.

### 4.2. Accuracy of GP-Canary Atlas

In preschool-age children, the GP-Canary Atlas underestimates the BA for all genders, showing statistically significant differences (MD = 17.036 mos.; *p* < 0.001). This level of underestimation is considerably greater than that reported in several European studies. For example, Martrille et al. (2023) [60] found a significant underestimation using the GP Atlas in Caucasian children from Southern France, with a mean difference (MD) of 1.27 mos. (SD = 1.56 mos.; *p* < 0.05). Similarly, Santoro et al. (2012) [43] reported underestimations in a Southern Italian cohort, with an MD of 1.2 mos. for boys (SD = 15.6 mos.; *p* = 0.18) and 4.8 mos. for girls (SD = 12.0 mos.; *p* < 0.001). Also, Kullman (1995) [45] also noted a smaller mean underestimation (MD = 4.8 mos.) in Swedish children.

The GP-Canary Atlas may lack accuracy in assessing the BA in preschool-age children due to several reasons. Firstly, during this period, known as “turgor primus”, children experience rapid and significant growth influenced by thyroid hormones, leading to high variability in ossification points that the Atlas evaluates. This variability makes it challenging to capture changes accurately. Secondly, the Atlas may not be well designed to reflect the developmental changes influenced by genetic and familial factors [61] rather than following a uniform pattern [62]. Additionally, the presence of children with constitutional delay of growth and puberty (CDGP) could introduce further growth variations that the Atlas may not capture due to insufficient calibration [63]. Finally, errors in reference PA-HW radiographs may also contribute to the lack of accuracy, suggesting that the Atlas might not be reliable for evaluating the BA in preschool children.

For school-age children, the GP-Canary Atlas also underestimates the BA but less than that observed in preschoolers, with a mean difference (MD) of 8.165 mos. (*p* < 0.001). The Atlas is more accurate for school-age girls than boys, largely due to a significant reduction in the underestimation of BA for girls (MD = 3.949 mos.; *p* = 0.239). This closer alignment with the Atlas’s developmental stages leads to measurements that are within the normal accuracy range established for this age group, which is up to 12 mos. [2].

During this age, alternating changes in bone maturation, such as increases in length, or “proceritas prima” and “proceritas secunda”, and weight, or “turgor secundus”, occur. In girls, these changes may be accelerated by early puberty [64,65], which is often associated with lifestyle factors, exposure to endocrine disruptors, or genetic determinants [66,67]. Obesity is a significant factor contributing to early puberty, particularly among Hispanic girls [68,69,70]. The girls in our sample have a high body mass index (BMI = 22.76; SD = 5.49), indicating obesity, which likely leads to an earlier onset of puberty and adolescence. In this phase, bone maturation becomes more regular and standardized compared to preschool children, resulting in earlier developmental stages for girls than boys. This reduces individual differences in bone growth patterns, making them more consistent and predictable, thereby allowing the GP-Canary Atlas to provide more accurate assessments of the BA in girls compared to boys.

With respect to teenagers, it has been demonstrated that the GP-Canary Atlas increases its accuracy as children mature. However, the Atlas slightly overestimates the BA with a mean difference (MD) of 3.159 mos. overall (*p* = 0.823), 4.497 mos. for girls (*p* = 0.980), and 4.85 mos. for boys (*p* = 0.095), though these overestimations are not statistically significant. This trend is consistent with studies in geographically similar regions, such as Portugal and Spain, where the GP Atlas also showed a progressive overestimation of the BA. For instance, Santos et al. [41] reported an increasing MD from 2 to 7 mos. in Portuguese adolescents, while comparisons with the Spanish-adapted Ebrí method showed overestimations ranging from 5 to 6.5 mos. (both *p* < 0.05) [71].

Other studies across Europe, including in Lower Saxony, Germany (Schmidt et al., 2007) [39] and the Loire Valley, France (Zabet et al., 2015) [55], demonstrated similar overestimations of the BA in teenagers when using the GP Atlas, with MDs ranging from 2.29 to 5.8 mos. (all *p* < 0.05). In Anglo-Saxon countries, Hackman and Black [37] found BA overestimations ranging from 1.62 to 11.05 mos. in adolescents aged 13 to 14 years in the Northern UK (*p* < 0.05), while Paxton et al. [34] observed an underestimation of 0.81 mos. in early childhood (*p* = 0.719) but a significant overestimation of 3.8 mos. in adolescence (*p* = 0.001) in Caucasian Australian children. Similar trends have been reported in Middle Eastern countries. For example, Soudack et al. (2012) [72] found significant underestimations in Israeli Caucasian children across various age groups: 6–10 years (MD = 2.3 mos.; *p* < 0.0001), 10–15 years (MD = 5.4 mos.; *p* < 0.0001), and 15–18 years (MD = 3.7 mos.; *p* < 0.0001). However, a slight overestimation was noted in those over 18 years (MD = 2.9 mos.; *p* = 0.0043). Similarly, Cantekin et al. (2012) [73] reported comparable results in Turkish Caucasian children, with underestimations in the range of 1.32 to 5.76 mos. (*p* < 0.05) in the 7–10-year-old age group and overestimations of up to 9 mos. (*p* < 0.05) in the 10–17-year-old age group.

Furthermore, the GP-Canary Atlas appears more accurate from puberty onwards compared to children from the nearby African continent. However, due to limited data, direct comparisons with North African regions influenced by the Berber ethnic group, the ancestors of the Canary Islands’ Guanches, are not possible. In other African countries, Tsehay et al. (2017) [74] reported that the GP Atlas overestimated the BA in children aged 10 to 22 years, with a mean difference (MD) of 8.7 mos. for males and 11.8 mos. for females (both *p* < 0.05). Similarly, Olaotse et al. (2023) [46] found overestimations in Botswana ranging from 3 mos. in early bone development to 11.2 mos. for adolescents aged 15 to 18 years (*p* < 0.05). Kowo-Nyakoko et al. (2023) [75] also reported that the GP Atlas overestimated the BA by approximately 9.12 mos. in peripubertal children in Zimbabwe.

At this period of development, the GP-Canary Atlas shows increased accuracy in predicting the BA for both boys and girls during the final phase of development, known as “turgor tertius”, which is characterized by rapid growth and hormonal changes driven by sex steroids. This is followed by the “post-pubertal period” or “internubil-puberal of Godin” marked by the closure of the epiphyseal growth plates, indicating the end of bone growth and the attainment of full skeletal maturity. In this phase of childhood, the differences in development between boys and girls decrease, leading to more synchronized and predictable maturation patterns. The GP-Canary Atlas captures this synchronization, as evidenced by the mean differences in BA for females (MD = −4.497 mos.) and males (MD = −4.85 mos.). These consistent changes allow the Atlas to predict the BA accurately within the normal range of up to 24 mos. [2], making it a reliable tool for assessing the BA in adolescents across genders.

## 5. Conclusions

This study confirms that the GP-Canary Atlas is a valid diagnostic tool for assessing BA in the pediatric population of the Canary Islands, demonstrating high intra-rater reliability and good inter-rater precision. However, the accuracy of the Atlas varies across developmental stages, with significant underestimations in preschool- and school-age children and slight overestimations in adolescents. Future works should focus on developing and validating age-adjusted versions of the Atlas to address these discrepancies as well as conducting additional studies to assess its applicability in diverse populations and clinical settings.

## Figures and Tables

**Table 1:** Characteristics of sample. Abbreviations: BMI = body mass index, mos = mos., statistical significance. *p*-values lower than these thresholds indicate statistically significant deviations from normality.

	Stage	Gender	N	Mean	SD	Min	Max	*p*-Value
Age (mos.)	Preschool	Female	24	39.33	15.18	20.00	67.00	0.235
		Male	45	46.49	13.33	18.00	69.00	0.105
	Scholar	Female	40	92.00	26.08	85.00	118.00	0.310
		Male	62	100.16	20.33	75.00	109.00	0.089
	Teenager	Female	16	144.17	23.81	102.00	168.00	0.150
		Male	27	151.53	20.17	107.00	192.00	0.080
Weight (kg)	Preschool	Female	24	14.52	2.05	9.80	18.60	0.215
		Male	45	13.09	2.17	7.40	18.00	0.175
	Scholar	Female	40	29.58	7.14	17.60	40.00	0.200
		Male	62	23.67	4.85	14.20	44.00	0.115
	Teenager	Female	16	33.84	4.62	22.00	39.50	0.250
		Male	27	34.21	3.19	23.80	45.70	0.140
Height (m)	Preschool	Female	24	0.91	0.07	0.77	1.05	0.289
		Male	45	0.94	0.05	0.80	1.10	0.175
	Scholar	Female	40	1.14	0.07	0.99	1.30	0.200
		Male	62	1.16	0.05	0.94	1.40	0.115
	Teenager	Female	16	1.33	0.04	1.21	1.37	0.250
		Male	27	1.33	0.03	1.16	1.45	0.140
BMI (kg/m^2^)	Preschool	Female	24	17.53	2.47	8.32	19.49	0.180
		Male	45	14.81	2.45	18.81	18.87	0.120
	Scholar	Female	40	22.76	5.49	13.45	20.29	0.175
		Male	62	17.59	3.60	12.57	20.92	0.150
	Teenager	Female	16	19.13	2.66	15.02	21.73	0.240
		Male	27	19.33	1.80	14.61	20.99	0.130

**Table 2:** Intra-rater agreement by time of measurement and gender. This table shows mean BA values, intra-class correlation coefficient (ICC), and 95% Confidence Interval (CI) for lower and upper limits for each rater (Rater 1, Rater 2, and Rater 3) at two different times of measurement (T1 and T2) for both female and male participants.

Group	Time of Measurement	Gender	Mean	ICC	95% CI Lower	95% CI Upper
Rater 1	T1	Female	77.65			
		Male	78.33			
	T2	Female	75.25	0.995	0.990	0.998
		Male	76.21	0.996	0.992	0.998
Rater 2	T1	Female	74.10			
		Male	82.47			
	T2	Female	70.57	0.990	0.979	0.995
		Male	80.94	0.992	0.982	0.996
Rater 3	T1	Female	78.79			
		Male	78.62			
	T2	Female	80.67	0.921	0.832	0.964
		Male	81.83	0.976	0.947	0.989

**Table 3:** Inter-rater agreement of BA assessment using GP-Canary Atlas by gender. This table presents mean BA values, intra-class Correlation Coefficient (ICC), and 95% Confidence Interval (CI) for lower and upper limits of agreement between different pairs of raters (Rater 1 vs. Rater 2, Rater 1 vs. Rater 3, and Rater 2 vs. Rater 3) for both female and male participants.

Groups	Gender	Mean	ICC	95% CI Lower	95% CI Upper
Rater 1–Rater 2	Female	75.73			
		72.34	0.982	0.968	0.990
	Male	78.33			
		81.70	0.944	0.902	0.968
Rater 1–Rater 3	Female	75.73			
		79.73	0.463	0.216	0.654
	Male	78.33			
		80.22	0.408	0.145	0.618
Rater 2–Rater 3	Female	72.34			
		79.73	0.509	0.273	0.688
	Male	81.70			
		80.22	0.327	0.052	0.557

**Table 4:** Accuracy of BA assessments using GP-Canary Atlas. Abbreviation: BA = bone age, CA = chronological age, MD = mean difference CA–BA, SD = standard deviation; W = Paired Samples Test or Wilcoxon signed-rank statistic. Statistical significance: (***) *p* < 0.001.

Stage		Mean	SD	MD	W	Z	*p*
Preschool (n = 69)	CA	43.485	14.476				
	BA	26.449	15.409	17.036	2297.5	6.517	<0.001 ***
Female	CA	39.331	15.182				
	BA	24.250	16.896	15.081	390.0	3.730	<0.001 ***
Male	CA	46.496	13.333				
	BA	31.598	24.881	14.898	776.0	4.920	<0.001 ***
Scholar (n = 102)	CA	95.684	23.906				
	BA	87.519	35.572	8.165	3306.5	3.346	<0.001 ***
Female	CA	92.001	26.086				
	BA	88.052	37.203	3.949	849.0	1.182	0.239
Male	CA	100.168	20.338				
	BA	86.870	33.876	13.298	829.0	3.898	<0.001 ***
Teenager (n = 43)	CA	148.883	23.665				
	BA	152.042	29.943	−3.159	339.00	−0.954	0.823
Female	CA	144.170	23.810				
	BA	148.667	24.231	−4.497	69.0	0.052	0.980
Male	CA	151.53	20.176				
	BA	156.38	18.179	−4.85	91.50	−1.686	0.094

## Data Availability

Data are contained within the article.
